# The Use of Rivaroxaban for Unprovoked Pulmonary Embolism in the Setting of Antithrombin Deficiency

**DOI:** 10.7759/cureus.8560

**Published:** 2020-06-11

**Authors:** Oscar J Gryn, Trivian Nguyen, Daniela Frankova

**Affiliations:** 1 Internal Medicine, Des Moines University, Des Moines, USA; 2 Internal Medicine, Mercyone Medical Center, Des Moines, USA

**Keywords:** antithrombin deficiency, rivaroxaban, venous thromboembolism (vte), pulmonary embolism (pe), direct oral anticoagulant therapy

## Abstract

A 24-year-old woman with antithrombin (AT) deficiency presented with right-sided pleuritic chest pain of five days duration with diagnosis of pulmonary embolism (PE) made at an outside hospital. After discussion of treatment options with the patient, her treatment was changed to rivaroxaban protocol. The case illustrates an appropriate treatment plan for patients with AT deficiency presenting with unprovoked PE, especially when prioritizing ease of use.

## Introduction

Antithrombin (AT) deficiency is a disorder in which the affected individual has decreased AT activity. AT deficiency can be acquired, such as through urinary AT loss in renal failure and nephrotic syndrome, or hereditary with an approximate prevalence of 0.02%-0.2%. Predisposition to developing venous thromboembolism (VTE) results, with 50%-90% of those affected experiencing a VTE in their lifetime [1].

In this report, we demonstrate the use of rivaroxaban, an oral factor Xa inhibitor, to treat and prevent recurrence of VTE in a 24-year-old woman with AT deficiency who experienced her first episode of unprovoked pulmonary embolism (PE).

## Case presentation

A 24-year-old woman with known AT deficiency of unknown zygosity diagnosed 17 years prior presented to the outside hospital with worsening five-day right-sided pleuritic chest pain. Accompanying symptoms were radiating pain to the right shoulder and right neck, dyspnea, lightheadedness, cough, headache, and nausea. She denied any similar prior episodes. Her past medical history includes depression and gastroesophageal reflux disease. The patient was a 2.5 pack-year smoker who switched to vaping daily for the past five years. She has a mother and maternal grandmother with AT deficiency.

**Figure 1:**
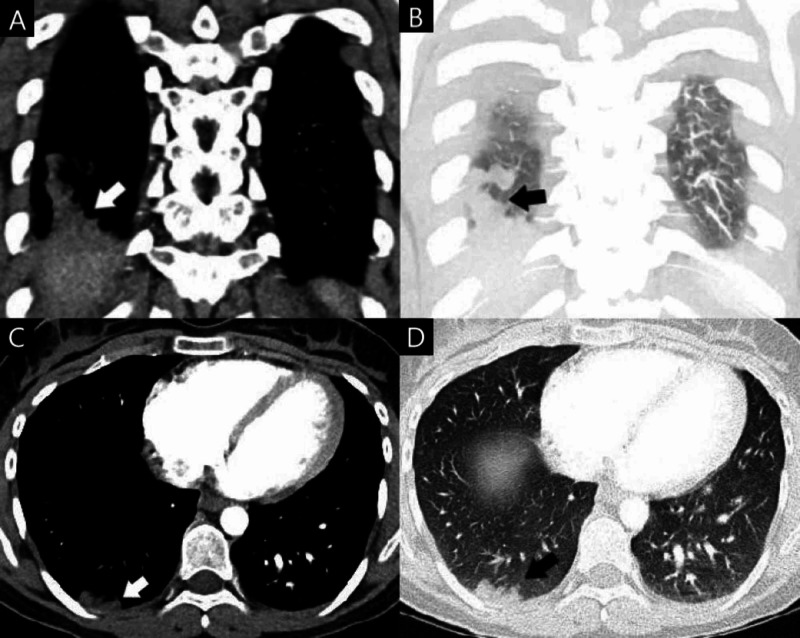
Contrast-enhanced coronal soft tissue window (A), coronal lung window (B), transverse soft tissue window (C), and transverse lung window (D) sections taken during evaluation at the outside hospital. Peripheral posterior right lower lung pulmonary infarct caused by acute pulmonary embolus of the right lower lung is visible.

She was initially seen at an outside facility the day prior with similar symptoms. There she was hemodynamically stable with normal laboratory findings. An electrocardiogram showed ventricular bigeminy with no ST-T wave abnormalities. CT angiogram of the chest showed acute pulmonary embolus of right lower lung with likely peripheral posterior right lower lung pulmonary infarct, as well as an indeterminate right upper lung pulmonary nodule and right hilar adenopathy (Figure 1). Ultrasound revealed no deep venous thrombosis (DVT) of either lower extremity. Echocardiogram was negative for right ventricular strain. She initially received intravenous heparin after evaluation in the ED and was discharged on warfarin; however, the patient sought second opinion at our hospital the following day.

Upon presentation to our institution, she complained of the same pleuritic chest pain. The patient agreed to begin life-long anticoagulation with a direct oral anticoagulant (DOAC) after education. She was discharged with rivaroxaban 15 mg twice daily with a plan to switch to 20 mg once daily after 21 days. On follow-up three months later, the patient endorsed good compliance with rivaroxaban with no complications since initiation of therapy.

## Discussion

The present case illustrates novel therapeutic opportunities for hemodynamically stable patients with their first VTE in the context of pre-existing AT deficiency. Historical standard of intravenous heparin with oral warfarin is contemporarily replaced by DOACs as a desirable alternative [2]. We chose rivaroxaban therapy, indicated in our case for use by FDA for treatment of pulmonary embolus and subsequent reduction in the risk of recurrence of DVT and PE.

**Figure 2:**
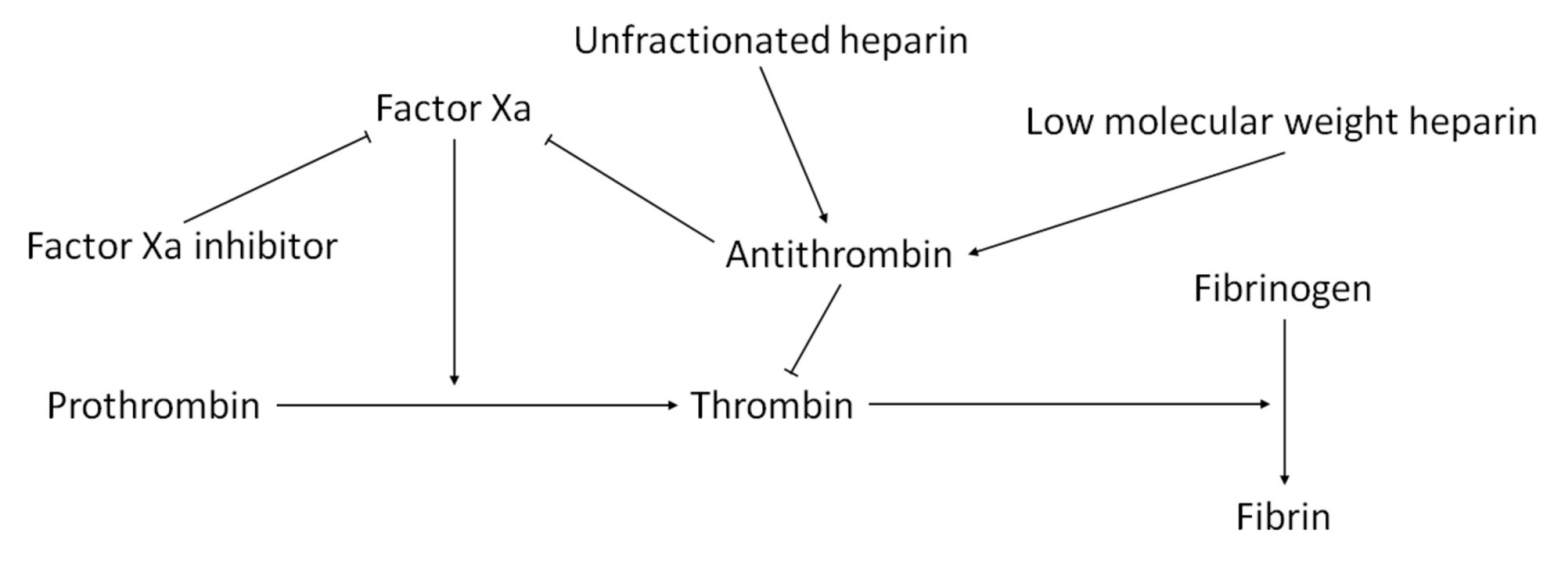
A selected portion of the coagulation cascade with featured anticoagulants. Factor Xa inhibitors such as rivaroxaban bind to and directly inhibit factor Xa activity. Antithrombin inactivates factor Xa and thrombin. Unfractionated and low molecular weight heparin potentiate antithrombin activity.

The AT deficiency represents a challenge in heparin dosing. In patients with low AT activity, higher doses of heparin would be required to produce the same desired effects as in a patient with normal AT activity, whereas DOACs such as rivaroxaban act independently of AT levels (Figure 2). Concentrated AT represents another strategy to augment the efficacy of heparin use in the setting of AT deficiency; however, currently there is no evidence demonstrating its efficacy in this context, and its high cost proves to be another limiting factor to its applicability [3]. Furthermore, DOACs may have a faster effect on anticoagulation than heparin plus warfarin [4].

Nonetheless, more studies concerning potential adverse effects of DOAC use in the setting of thrombophilia should be ascertained. A meta-analysis comparing the efficacy and safety of four DOACs (rivaroxaban, dabigatran, apixaban, and edoxaban) to warfarin conducted by van der Hulle et al. found similar efficacy with a slightly lower risk of bleeding complications with DOAC use [5]. Adverse effects of DOACs were reported following initiation of rivaroxaban in two patients with protein S deficiency [6]. Otherwise, no other complications were seen on follow-up in patients with a thrombophilia treated with a factor Xa inhibitor, with no adverse effects noted in any patients with AT deficiency [7-11].

In the present case, our patient likely did not achieve therapeutic international normalized ratio (INR) at the outside facility as she was discharged one day after admission. When utilizing IV heparin with oral warfarin, the standard of care involves attaining an INR of two to three before discharge, which likely did not occur as this takes five to six days on average after starting warfarin [12]. After discussion of anticoagulation options with the patient when she presented to us, we recommended rivaroxaban because of its ease of use, minimal drug or diet interactions, and good potency in the context of AT deficiency [4]. Nevertheless, further studies are needed to determine the optimal dose of DOACs such as rivaroxaban in patients with AT deficiency for prevention of VTE recurrence.

## Conclusions

Herein, we describe the case of an unprovoked PE and subsequent management with rivaroxaban protocol in a patient with known AT deficiency. Current popular options for life-long anticoagulation treatment include IV heparin plus oral warfarin or a DOAC. This case illustrates that the use of DOACs for such a presentation represents a safe, desirable alternative to the historical standard of IV heparin with oral warfarin, especially when considering the possible disease-drug interactions of the latter therapy with AT deficiency.
